# A prospective randomised comparative study of three palliative radiotherapy schedules in incurable locally advanced head and neck cancer

**DOI:** 10.3332/ecancer.2025.2021

**Published:** 2025-10-22

**Authors:** Shivani Malik, Ashok Kumar Arya, Renu Singh, Lalit Raj, Aditya Uttam

**Affiliations:** 1 Radiation Oncology Department, Sarojini Naidu Medical College, Agra 282003, Uttar Pradesh, India; 2 Radiation Oncology Department, KGMU, Lucknow 226003, India

**Keywords:** locally advanced, head and neck cancer, squamous cell carcinoma, palliative radiotherapy, Quad shot, Christie, conventional

## Abstract

**Introduction:**

There are multiple dose fractionation schedules for palliative radiotherapy (RT) of patients belonging to incurable locally advanced head and neck squamous cell carcinoma (LAHNSCC), but none is accepted as standard of care. In this study, we have compared three palliative RT schedules: Quad shot versus Christie versus conventional.

**Aim and objectives:**

To assess and compare the toxicity, tolerance and effectiveness of three palliative RT regimens for incurable LAHNSCC.

**Methods:**

A total 105 patients were randomised equally 1:1:1 in 3 arms with 35 patients each. Arm A: 14.8 Gy in 4 fractions with 3.7 Gy per fraction in 2 fractions per day 6 hours apart for 2 consecutive days for 3 sessions at 3 weekly interval. Total dose was 44.4 Gy in 12 fractions over 6.2 weeks. Arm B: 50 Gy in 16 fractions over 3.1 weeks with 3.125 Gy per fraction with 5 fractions per week.

ARM C: 20 Gy in 5 fractions with 4 Gy per fraction with 5 fractions per week in 2 sessions at 3 weekly interval. Total dose was 40 Gy in 10 fractions over 3.5 weeks. All the patients were assessed pre RT, post RT and at 1 month follow up.

**Results:**

All three arms had similar baseline characteristics 80% patients in arm A, 80% in arm B and 72% in arm C completed intended treatment. Drop out rate was 20%, 20% and 28%, respectively. Defaulters were excluded from the study. Mean ± standard deviation scores of individual domains of quality of life (QOL) (pre and post RT) have been calculated. Tumour response was partial in 92.8% patients of Arm A, 92.8% of Arm B, 96% of Arm C. Complete nodal response was seen in 71.4% patients of Arm A, 85.7% of Arm B, 60% of Arm C with Grade 3 mucositis observed in only 1 patient of Arm A and 4 patients of Arm B. Median overall survival was highest in Arm B, i.e., 6 months.

**Conclusion:**

Statistically significant objective response and improvement in all QOL parameters, along with performance status, was observed in all the 3 arms. However, Christie arm patients had more symptomatic relief with better loco-regional control and prolonged overall survival as compared to other arms with acceptable toxicities. Therefore, as per this study, Christie schedule may be considered for adequate palliation of incurable LAHNSCC patients.

## Introduction

Globally, head and neck cancers (HNCs) are among the most common malignancies [1], accounting for approximately 1,094, 000 new cases of lip and oral cavity cancer alone in 2022 [1, 2]—making it the 16th most common cancer site worldwide [2]—while total HNC (including oropharynx, larynx, hypopharynx, nasopharynx, salivary glands and thyroid) contribute substantially to the global cancer burden [3]. In India, lip and oral cavity cancer ranked 2nd among all cancers in 2022, with about 143, 759 new cases and 79,979 deaths [4]. Overall, India recorded approximately 1.46 million new cancer cases in 2022, with one in nine people likely to develop cancer in their lifetime [5]. While GLOBOCAN does not provide a combined figure for locally advanced head and neck squamous cell carcinoma (LAHNSCC), studies using GLOBOCAN data suggest that advanced disease (unresectable or incurable at presentation) comprises around 70%–75% of HNC cases in India [6]. Ideal management for locally advanced HNC is a multi-modality approach involving all three disciplines of oncology, i.e., surgery, radiation and chemotherapy. Locally advanced lesions that are inoperable and incurable (stage IVA and IVB with bulky disease) are best treated with palliative chemotherapy and/or radiotherapy (RT) and/or best supportive care. With more advanced lesions and poor performance status, surgery is generally not feasible. Chemotherapy has shown little efficacy in the palliation of bulky head and neck cancer lesions. Hypofractionated RT is very beneficial in patients with advanced solid tumours for good loco regional control, resulting in symptomatic relief within a short span of time, along with minimal toxicities. Due to the poor general condition of these patients and the incurable nature of the disease, many are not candidates for aggressive multimodal radical chemo RT or prolonged palliative treatment. In such cases, a short-course or moderate course treatment approach proves to be an effective palliative option for loco-regional control, alleviate distressing symptoms and enhance patient’s quality of life (QOL) with manageable side effects. Additionally, this approach aims to balance treatment efficacy with economic considerations, reducing treatment duration, hospital stays and machine load, ultimately optimising healthcare resource utilisation.

Despite sufficient evidence on the benefits of RT in advanced head and neck squamous cell cancer. There is no standard dose fractionation schedule of radiation therapy for palliative treatment of locally advanced inoperable and incurable HNC. The various authors have adhered to various schedules for the total dosage and fractional amounts of treatment in order to obtain justification in locally advanced head and neck carcinoma (LAHNC).

In order to determine the best standard palliative radiation treatment for inoperable and incurable LAHNSCC, the current study aims to assess and compare the toxicity, tolerability and effectiveness of three different palliative RT regimens.

## Methodology

The prospective randomised study was conducted on 105 patients from 1st March 2023 to 30th March 2025 on treatment naïve patients of LAHNSCC who were not suitable for definitive treatment after obtaining informed consent from each patient. Sample size in the present study was calculated by a statistician of Department of Community Medicine, S N Medical College, Agra, using Mean and Standard Deviation of QOL in software ‘*p* value: a Stastical Tool’.

{Quad shot = 60+_ 23.77

Christie = 40+_24.16

Conventional = 60+_21.87}

Calculated sample size = 28+4 = 32 (with drop out rate of 15%.) [15]

Total calculated sample size = 32* 3 = 96, with reference to the first open-label study comparing these three palliative RT schedules done by Soni *et al* [14].

So, a few more patients were taken taking into account the default rate in his study, leading to 105 patients in total in the present study.

A simple randomisation of 1:1:1 by draw of lots for the division of 105 patients into three arms was followed, resulting in 35 patients in each arm.

Inclusion criteria for the patient selection in the study were: Karnofsky Performance Status >40; Complete haemogram with platelet count >100,000/mm^3^, total leukocyte count (TLC) >4,000/mm^3^ and Hb>8 gm/dL; Renal function tests with serum creatinine <1.5 mg/dL and blood urea <40 mg/dL; Liver function tests (SGOT <35 IU/L, SGPT <40 IU/L; AJCC stage IV with a positive biopsy for head and neck squamous cell carcinoma that is not compatible to definitive surgery, chemoradiotherapy or radiation therapy. Patients with any of the following conditions were excluded from the study: Distant metastases; previously received chemotherapy, surgery or radiation therapy for the disease; breastfeeding or pregnant; associated medical problem, such as liver, heart or kidney disease; Individuals with a primary in the thyroid, paranasal sinuses, salivary glands, nasal cavity or nasopharynx; Histopathology other than squamous cell carcinoma. 

Arm A (Quadshot): 35 patients received a radiation dosage of 14.8 Gy divided into 4 fractions at a rate of 3.7 Gy each fraction, with two fractions per day separated by 6 hours for 2 consecutive days. At 3-week intervals, the identical strategy was repeated for two more sessions. Over 6.2 weeks, a total of 44.4 Gy was given in 12 fractions. The replanning process used spinal cord sparing for the last of the two fractions.

Arm B (Christie): 35 patients received a radiation dose of 50 Gy in 16 fractions over a period of 3.1 weeks at the rate of 3.125 Gy per fraction with 5 fractions per week. Spinal cord sparing was done after 11 # in replanning.

ARM C (Conventional): 35 patients received radiation dose of 20 Gy in 5 fractions at the rate of 4Gy per fraction with 5 fractions per week. The same plan was repeated for one additional session at 3 weekly interval. During 3.5 weeks, a total of 40 Gy was given in 10 fractions. For the final fraction, spinal cord sparing was done in the replanning.

Patients were treated in the supine position after a pre treatment simulation to determine the boundaries of the field, which included the neck nodes, disease extension and the main tumour. Parallel opposing fields were used to treat the patients and the dose was directed toward the midplane at the centre axis by using Cobalt-60 teletherapy.

Every patient had a thorough history, a general physical examination and a systemic examination as part of the pre treatment evaluation. Karnofsky Performance Status (KPS) was used to evaluate the general condition. A complete haemogram comprising haemoglobin, platelet count, TLC, differential leukocyte count and peripheral blood film was used for the haematological evaluation. Blood urea, serum creatinine, SGOT and SGPT levels were estimated as part of a biochemical evaluation to evaluate the kidney and liver functions. All patients had radiological evaluation, which included abdominal and pelvic ultrasonography, a chest X-ray, and a lateral view of the soft tissues of the neck with dental examination. For precise staging, a face and neck computed tomography scan was performed. The patients were staged in accordance with the American Joint Committee on Cancer's eighth edition.

Every patient in the trial was consistently evaluated from the beginning of treatment, both daily during treatment and once a week during scheduled breaks. By carefully examining each patient for local disease state and monitoring for acute toxic side effects of radiation, a comprehensive clinical evaluation of each patient's tolerance to the administered treatment was completed. The Radiation Therapy Oncology Group (RTOG) and World Health Organisation (WHO) toxicity guidelines were used to evaluate radiation effects. From the first day of radiation treatment until day 90, RTOG acute morbidity scoring criteria were applicable. After that, the RTOG criteria for late effects were applied. WHO response criteria were used to evaluate objective response (primary and nodal response). The University of Washington quality of life (UWQOL) questionnaire version 4.0 was used to measure QOL. The Kaplan–Meier survival estimate was used to evaluate both overall survival and progression-free survival.

At the end of treatment, all patients were evaluated to identify any acute side effects, such as skin reactions, mucositis or dysphagia.

Every patient received routine OPD follow-up for a minimum period of 6 months or until death. Clinical evaluation for treatment-related problems, any indications of distant metastases, and thorough local examination for loco-regional disease control were done at each visit. The QOL was evaluated at the start of treatment, on the day of treatment completion and 1 month after the planned course of treatment was finished.

The study's findings about QOL, safety, tolerability, toxicity and response in each arm have been documented.

### Statistical analysis

To determine differences across all groups in terms of tumour and nodal response, treatment-related toxicities, performance status improvement, overall survival and QOL, the resulting data were evaluated, processed and compared using SPSS VERSION 28.0.1. Comparison between categorical measurements was done by using chi square test. Wilcoxan signed Rank test was used to statistically analyse the QOL assessment.

## Results

Out of 105 patients enrolled in the study, with 35 in each arm, 7 patients (20%) defaulted treatment in ARM A, 6 patients (17.1%) in ARM B and 10 patients (28.5%) in ARM C. So, the drop out rate was variable across the three arms. In Arm A, 4 patients left the treatment after completing 1st session and 3 patients defaulted after completing 2 nd session. 6 among the defaulters were females who did not complete the intended treatment due to family issues (ignorance by family members, financial, trust issues in allopathic medicine and having more belief in ayurvedic care, illetracy) and one was the eldest patient of the study who left because of poor general condition. In Arm B, 5 patients were left out due to significant mucositis and 1 patient expired in ARM B during treatment after receiving 1 # (death not related to treatment). In Arm C, 8 patients defaulted after completing 1st session as they did not feel any change in their disease status and 2 patients left out after 3 fractions due to a conveyance problem. These patients were not excluded from the study to minimise the attrition bias. All the patients were treated as per intention-to-treat analysis.

Median age of the study population was 52 years (30–79), 50 years (27–70), 53 years (22–73) with male predominant population of 85.7%, 91.2%, 71.4% in ARM A (Quadshot), B (Christie) and C (Conventional), respectively. The majority of the patients had addiction to chewable tobacco (85.7% in Arm A and Arm B, 80% in Arm C), followed by smoking and then alcohol. Buccal mucosa was the most common site involved (54.3%, 34.2% and 45.7%) with MDSCC being the major histology (74.2%, 65.7% and 62.8%) in ARM A, B and C, respectively. All the patients (100%) belonged to stage IV, with the majority in stage IV A in all the arms. The demographic profile has been depicted in Table 1.

The most common symptom was pain, followed by difficulty in swallowing, poor appearance, with other symptoms of depressed mood and anxiety, difficulty in chewing and decreased physical and social activity. Ryles tube insertion prior to initiation of RT was required in tumours of oropharynx and oral cavity where the presenting symptoms were dysphagia. Otherwise, it was inserted at symptom aggravation. In this study, nasogastric tube placement was required in 47% patients at some point of treatment. Tumour stage alone was not the sole deciding factor for recruiting a patient under this study. Performance status, co-morbidities, expected survival, socio-economic factors were all taken into consideration.

Patients were assessed on the basis of KPS before and after completion of treatment in all the arms. There was statistically significant improvement of KPS Score Post RT in all the arms, *p* value <0.0001.

(Arm A: 47.86^+^___9.17 to 63.93^+^___ 7.86, Arm B: 53.45^+^___ 8.14 to 69.63^+^_ 6.49 and Arm C: 50.8^+^___11.52 to 64.0^+^___9.57) However, maximum enhancement of performance status was seen in ARM B as depicted in Table 2.

Mucositis and Dermatitis were the most common toxicities experienced by the patients after radiation. Most commonly, mucositis was observed after 8# of RT in all the arms. During RT, Grade 3 mucositis was observed in 1 patients of Arm A, 4 patients of Arm B. No case of Grade 3 dermatitis was seen in any of the arm. 20 patients were hospitalised for mucositis management. Post RT, Grade 3 mucositis occurred in 1 patient of Arm A and 4 patients of Arm B. Grade 3 dermatitis was not observed in any of the arm. No significant toxicity was observed in any of the arm at 1st follow up after 1 month of RT completion.

An objective locoregional response evaluation was conducted on the day when the therapy was finished. None of the patient had a complete tumour response in any of the arm. 92.8%, 92.8%, 96% patients in Arm A–C, respectively, had partial tumour response. 7.2%, 7.2% and 4% patients in Arm A–C, respectively, had stable disease (SD). None of the patients in any of the arms developed progressing disease. 75%, 85.7%, 76% patients had complete nodal response in Arm A–C, respectively, while 25%, 14.3% and 24% had partial nodal response. Complete nodal response was seen maximum in Arm B. For tumour response, both ARM A and B were equivalent as depicted in Table 3. The patients were followed for a minimum period of 6 months (range 6–21 months) or until death. 9 patients lost to follow up in Arm A, 8 in Arm B and 10 in Arm C at variable lengths of time.

**Figure 1.**
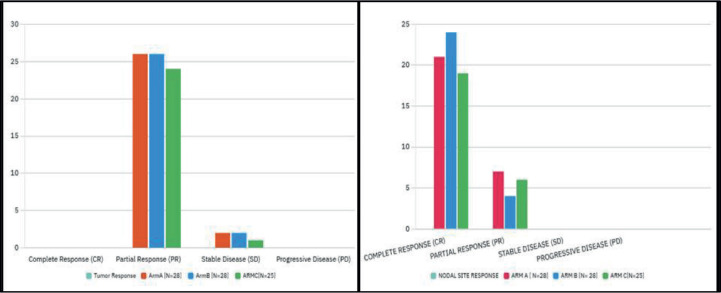
Bar chart depicting objective response across all arms.

26 patients in Arm A, 27 in Arm B, 25 in Arm C came for 1st follow up accounting for 78 patients in total. 2 patients in Arm A, 1 in Arm B, expired after RT in 1st month. After 1st follow up, 12 patients in Arm A, 12 in Arm B and 14 in Arm C were put on i/m methotrexate 50 mg weekly along with 250 mg daily dose of Geftinib. 3 patients in Arm A, 1 in Arm B were put on paclitaxel-carboplatin combination chemotherapy. After receiving RT, 42 patients were able to tolerate palliative chemotherapy as they had an improvement in their generalised status. Patients were put on different chemotherapy regimens after radiation as per the performance status of the patient to control disease progression, to improve symptomatic relief, survival prolongation, to address systemic disease and control micro metastasis, to delay further re irradiation or any invasive intervention and as a part of multimodal palliative intent (Figure 1).

In the present study, using UWQOL questionnaire version 4, the mean ± standard deviation score of patients before treatment and after completion of treatment in all the arms was assessed. The symptoms at presentation and after treatment were documented. As patients were in the advanced stage of disease main complaints were pain, unpleasant appearance, difficulty in swallowing, difficulty in chewing and hoarseness of voice. In overall QOL, there was significant improvement in all the arms (*p* value <0.001) with values of (22.54+-1.64 to 68.78+-19.68) in ARM A, (21.89+-12.45 to 71.48+-19.88) in ARM B, (22.65+-68.64 to 18.64) in ARM C. There was substantial improvement in pain, appearance, swallowing, chewing, speech, activity, recreation, mood and anxiety. Score declined for taste and saliva in all the arms significantly. Pain score improved maximum in Christie arm from 21.43+-16.27 to 89.29+-14.32. Appearance score improved maximum in ARM A from 40.18+-19.65 to 83.93 +-15.34. The shoulder domain score remained unchanged in all the arms. In the rest of the domains, the pattern of improvement was similar in all the arms. Symptomatic relief was maximum in ARM B. A detailed description of each domain score has been mentioned in the Table 4.

Median overall survival was 4 months in the Quadshot arm, 6 months in Christie arm and 3.5 months in the conventional arm, as shown in Table 5.

## Discussion

To the best of our knowledge, this is the second open label, prospective randomised study comparing three palliative RT schedules for the management of LAHNC. The study primarily aimed to alleviate distressing symptoms, including painful ulcers, pain and difficulty swallowing, while also evaluating treatment-related toxicities and overall QOL. Given that most of our patients come from lower socioeconomic backgrounds and present at an incurable stage, improving their QOL and considering cost-effectiveness are crucial aspects. Due to poor performance status and the unresectable nature of the disease, radical multimodal treatment is not a viable option for all patients. Instead, these advanced cases require palliative therapy and/or best supportive care.

**Figure 2.**
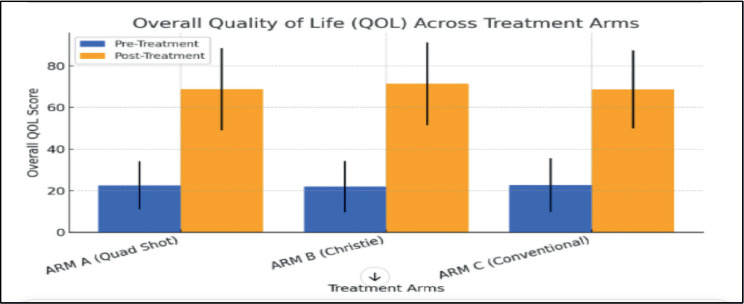
Bar chart depicting QOL pre and post RT.

**Figure 3.**
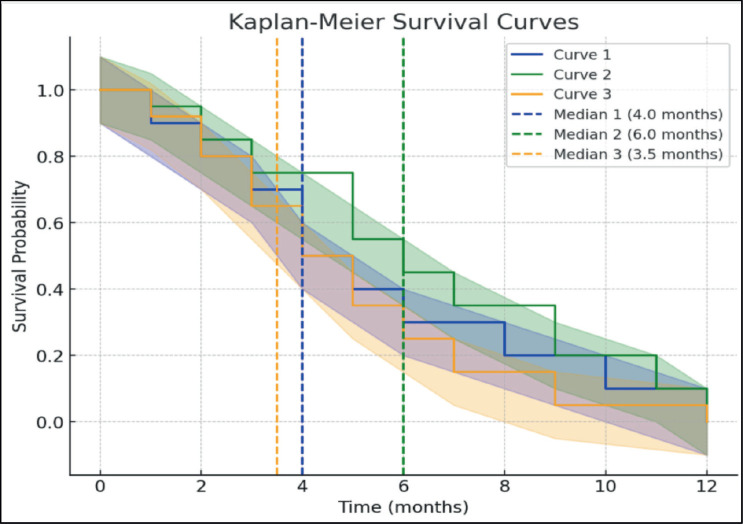
Kaplan Meir survival curve showing overall survival across all arms.

In a study by Mohanti *et al* [7] using 40 Gy/10#, none of the patient had grade 3 toxicity. Das *et al* [13] in their study using 40 Gy/10# reported grade 3 mucositis and dermatitis in 18% and 3% patients, respectively. In a study by Chen *et al* [10] using Quad Shot schedule, 9% patients had grade 3+ toxicity, which was considered significant. Ghoshal *et al* [11] in her study using Quad Shot schedule reported that none of the patient had grade 3 toxicity. Al -Mamgani *et al* [12] using Christie schedule reported that grade 3+ dermatitis and mucositis were observed in 45% and 65% patients, respectively. Srivastava *et al* [17] in her study using Christie regimen reported that grade 4 dermatitis and mucositis were encountered in 2.7% and 1.8% cases, respectively. Soni *et al* [14] stated that grade 3 dermatitis was observed in 23.3%, 40% and 20% with grade 3 mucositis 30.6%, 53.3% and 23.3% in arm A, B and C, respectively, accounting for the highest toxicity in Christie schedule. In the current study, toxicity was observed mostly after 8# of RT. Grade 3 mucositis was documented in 3.5%, 17.8% and 0% cases during RT in Arm A–C, respectively. 24.7% patients were hospitalised for mucositis management. Post RT; significant mucositis (grade 3) occured in 3.5%, 14.2% and 0% cases in Arm A–C, respectively. Mucositis was not significant in any of the arm at 1st follow up. No cases of grade 3 dermatitis were observed in any of the arm during any time. Similar to Soni *et al* [14], Christie schedule was associated with the highest number of cases with significant toxicity, but it was acceptable when compared to other studies in the literature and easily manageable (Figures 2 and 3).

Mohanti *et al* [7] in his study mentioned that 37% patients achieved partial response (PR). Agarwal *et al* [9] reported 10% patients had CR, while 73% had PR. Chen *et al* [10] reported a maximum response of 83% in Quadshot arm when compared to others. Ghoshal *et al* [11] reported that 50% patients had an objective response. Al-Mamgani *et al* [12] reported 62% locoregional control. Mudgal *et al* [15] concluded in his study that good objective response was seen in 82.6% and 84.7% of patients at primary and nodal sites, respectively. Soni *et al* [14] in his study reposted complete response (CR) rates were (30%, 43.3% and 26.6%), partial response was (53.3%, 36.6% and 50%, and no response rates were (16.6%, 20% and 23.3%) in Arm A–C, respectively. In the present study, CR at tumour site was not seen in any of the arm. Partial response rates were (92.8%, 92.8% and 96%) in Arm A–C, respectively. Only few patients had SD. Disease progression was not seen in any of the arm. Nodal response was CR in (75%, 85.7% and 76%), PR in (25%, 14.3% and 24%) in ARMA, B and C, respectively. Node was not stable or progressive in any of the arm. Christie arm had better locoregional control in terms of response. Locoregional control was also good in other arms when compared to the studies described in the literature. After 1st follow up, in view of residual disease 42.8% patients in ARM A, 42.8% in ARM B and 56% patients in ARM C were put on palliative chemotherapy as per institutional protocol in the form of I/M Methotrexate q weekly with Tab. Gefitinib 250 mg od. 10.7% patients in ARM A, 3.5% in ARM B were put on paclitaxel-carboplatibn combination chemotherapy. In total, 51.8% patients were able to tolerate palliative chemotherapy after improvement in their generalised state post RT. Patients were put on different chemotherapy regimens after radiation as per the performance status of the patient to control disease progression, to improve symptomatic relief, survival prolongation, to address systemic disease and control micrometastasis, to delay further re irradiation or any invasive intervention and as a part of multimodal palliative intent. The data were comparable to the study by Soni *et al* [14].

Soni *et al* [14] in his study reported that statistically significant improvements were observed in scores of pain, appearance, activity, recreation, mood, anxiety, physical domain score, social domain score, HRQOL 7 days and overall QOL (20+-1.51 to 60+-23.77) with reduction in taste and saliva score in the Quad shot arm. For the Christie regimen and conventional regimen also, pattern of improvement and reduction in score of domains was same with different values (20+-3.65 to 40+-24.16) and (20+-7.43 to 60+-21.87), respectively. Although, more improvement was observed in the Quadshot schedule in the physical domain, social domain, HRQOL7 days and overall QOL as compared to other arms. In a study by Ghoshal *et al* [11] psychosocial domain, HRQOL, overall QOL and almost all physical symptoms scores improved significantly. Das *et al* [13] also reported that there was an enhancement in all aspects of QOL. Kumar *et al* [16] reported that symptomatic relief was better in Quad shot arm. Al-Mamgani *et al* [12] better QOL of patients in Christie arm. QOL enhancement was also seen in a study by Corry *et al* [8] in quadshot arm. In the present study, there was significant improvement in overall QOL in all the arms (*p* value <0.001) with values of (22.54+-1.64 to 68.78+-19.68) in ARM A, (21.89+-12.45 to 71.48+-19.88) in Arm B, (22.65+-68.64 to 18.64) in ARM C. There was significant amelioration in pain, appearance, swallowing, chewing, speech, activity, recreation, mood and anxiety. Score declined for taste and saliva in all the arms significantly. Symptomatic refinement was maximum in Arm B. Most of the studies till now have reported pain as the commonest symptom, which was in accordance with this study. Pain score improved maximum in Christie arm from 21.43+-16.27 to 89.29+-14.32.

Das *et al* [13] observed survival of 7 months. Mudgal *et al* [15] stated that overall survival was 9 months. Mohanti *et al* [7] reported an overall survival of 13 months. Overall survival was 40% at 1 year in Al-Mamgani *et al* [12] study. Soni *et al* [14] reported median survival was 11.5 months in Arm A, 10.5 months in Arm B and C. In the present study, median overall survival was 6 months in Arm B, 4 months in Arm A and 3.5 months in Arm C. Arm B had the highest survival.

## Conclusion

Short-course palliative treatment shows encouraging outcomes in locally advanced head and neck cancer in all the arms, delivering a high dose rate to the primary site, providing significant symptom relief and causing minimal toxicity. Objective response was observed in all three arms. Statistically significant overall QOL improvement was observed in all three arms along with an enhancement in performance status. Significant toxicity was minimal in all the arms. Median overall survival of the patient also improved in all the arms when compared to patients who has not taken any treatment. However, patients in Christie arm had better loco-regional control, with improvement in QOL and performance status, along with the highest median overall survival as compared to other arms. Although significant toxicities were seen in a few patients but they were manageable. The Christie schedule offers better palliation of distressing symptoms like pain, dysphagia and ulceration. It provides more durable and clinically meaningful improvement in QOL, especially in patients with moderately good performance status. Many studies, including institutional experiences in India, have shown that patients on the Christie schedule report better post-treatment QOL scores despite initial toxicities. Mucositis is usually transient, while the relief from symptoms tends to last longer, leading to a net gain in overall well-being. It offers a better short-term local control of disease compared to ultra-short schedules like Quad Shot or single-fraction RT, which may lead to earlier symptom recurrence. This may reduce the need for re-irradiation or repeat admissions, especially beneficial in resource-limited settings. Although mucositis may be Grades 2–3 in many cases, it is generally manageable with supportive care. It strikes a good balance between intensity (tumour shrinkage) and tolerability, especially for patients with expected survival of 3–6 months or more. It is shorter than conventional fractionation but more effective than ultra-short regimens. It often fits well with hospital scheduling, resource availability and patient logistics making it very Feasible in Indian/Resource-Constrained Settings. Although nowadays, Quad shot has been noted as a well-known palliative schedule but as per this study; Christie schedule may be considered for adequate palliation of the patients of LAHNSCC.

## Limitations

These patients typically report a degraded performance status as a result of a persistent dietary deficit. Socioeconomic issues have also been a major reason for delaying or defaulting protracted courses of radiation treatment among our patients. Poor patient compliance to treatment, limited enrolment caused difficulties in outcome assessment. The limitation of this study is shorter follow-up period and small sample size. A larger multicentre trial with a longer follow-up period is required to validate the study's findings.

## List of abbreviations

HNC, Head and neck cancer; KPS, Karnofsky Performance Scale; LAHNC, Locally advanced head and neck carcinoma; LAHNSCC, Locally advanced head and neck squamous cell carcinoma; UWQOL, University of Washington quality of life.

## Conflicts of interest

The authors affirm that they have no conflicts of interest, either financial or otherwise.

## Funding

No direct or indirect funding was received for this clinical work.

## Ethical approval

SNMC/IEC/THESIS/2024/195. Ethical certificate was obtained from the Institutional Ethics Committee of SNMC Agra, date: 2 february 2024.

## Figures and Tables

**Table 1.** Demographic profile.

Age (years)	Arm A	Arm B	Arm C
Range	30–79	27–70	22–73
Median	52	50	53
Gender			
Male	30 (85.7%)	31 (91.2%)	25 (71.4%)
Female	5 (14.3%)	4 (11.4%)	10 (28.6%)
Addiction			
Smoking	20 (57.1%)	27 (77.1%)	24 (68.6%)
Tobacco	30 (85.7%)	30 (85.7%)	28 (80%)
Both	14 (40%)	13 (37.1%)	13 (37.1%)
Alcohol	7 (20%)	4 (11.4%)	2 (5.7%)
All	7 (20%)	3 (8.6%)	3 (8.6%)
Site			
Oral cavity	30 (85.7%)	21	27 (77.1%)
Orophayrnx	4 (11.4%)	11	4 (11.4%)
Larynx	1 (2.8%)	3	4 (11.4%)
Histology			
MDSCC	26 (74.2%]	23 (65.7%)	22 (62.8%)
PDSCC	7 (20.2%)	6 (17.6%)	6 (17.6%)
WDSCC	2 (5.41%)	6 (17.6%)	7 (21.2%)
Stage			
IV A	25 (71.4%)	23 (65.7%)	17 (48.6%)
IV B	10 (28.6%)	12 (31.3%)	18 (51.4%)

**Table 2.** KPS score evaluation pre and post RT.

Pre RT KPS score	ARM A	ARM B	ARM C
40	12 (42.8%)	8 (22.8%)	14 (4%)
50	11 (44.0%)	13 (37.1%)	8 (22.8%)
60	8 (22.8%)	11 (31.4%)	7 (20%)
70	4 (11.4%)	3 (8.5%)	6 (17.1%)
Mean	8.75+_ 3.59	8.75+_ 4.35	8.75+_ 3.59
Post RT KPS score	ARM A	ARM B	ARM C
40	3 (10.7%)	1 (3.6%)	5 (20%)
50	14 (50%)	8 (28.6%)	8 (32%)
60	8 (28.6%)	14 (50%)	9 (36%)
70	3 (10.7%)	5 (17.8%)	3 (12%)
Mean	63.93 +_ 7.86	69.63 +_6.49	64.0 +_9.57
*p* value	0.0001*	0.0001*	0.0001*

* = statistically significant value or result

**Table 3.** Objective response assessment.

Tumor response	ArmA	ArmB	ARMC
CR	0	0	0
PR	26 (92.8%)	26 (92.8%)	24 (96%)
SD	2 (7.2%)	2 (7.2%)	1 (4%)
Progressive disease (PD)	0	0	0
**Nodal response**			
CR	21 (75%)	24 (85.7%)	19 (76%)
PR	7 (25%)	4 (14.3%)	6 (24%)
SD	0	0	0
PD	0	0	0

**Table 4.** QOL assessment pre and post RT using Wilcoxan signed rank test.

Symptom	ARM A (Quad Shot)			ARM B (Christie)			ARM C (Conventional)		
	Pre RT	Post RT	*p* value	Pre RT	Post RT	*p* value	Pre RT	Post RT	*p* value
	Mean ± SD	Mean ± SD		Mean ± SD	Mean ± SD		Mean ± SD	Mean ± SD	
Pain	17.59 ± 11.63	80.56 ± 21.18	000*	21.43 ± 16.27	89.29 ± 14.32	000*	34.38 ± 14.39	89.67 ± 17.55	000*
Appearance	40.18 ± 19.65	83.93 ± 15.34	000*	38.39 ± 24.04	78.57 ± 25.19	000*	37.50 ± 14.74	77.08 ± 12.59	000*
Activity	38.39 ± 15.93	76.79 ±16.57	000*	38.39 ± 14.04	71.43 ± 22.27	000*	42.71 ± 15.60	66.67 ± 17.55	000*
Recreation	36.61 ± 15.93	68.75 ± 27.74	000*	48.21 ± 22.49	78.57 ± 18.89	000*	48.96 ± 11.61	69.79 ± 12.74	000*
Swallowing	34.36 ± 19.33	66.82 ± 20.39	000*	23.57 ± 15.18	56.07 ± 16.17	000*	38.71 ± 16.27	72.46 ± 15.99	000*
Chewing	16.07 ± 16.96	49.11 ± 35.01	000*	17.86 ± 17.82	51.14 ± 17.06	000*	19.79 ± 12.72	37.50 ± 22.12	000*
Speech	34.50 ± 26.56	73.96 ± 24.59	000*	27.32 ± 25.83	58.29 ± 26.83	000*	33 ± 0	61.33 ± 12.94	000*
Shoulder	2.96 ± 8.80	7.43 ± 15.42	0.096	8.25 ± 14.55	5.89 ± 12.87	0.573	1.32 ± 6.60	6.60 ± 13.47	0.043
Taste	45.07 ± 20.94	10.71 ± 22.38	000*	57.29 ± 15.64	25.93 ± 13.79	000*	41.54 ± 17.97	8.25 ± 14.59	000*
Saliva	19 ± 24.77	4.71 ± 11.76	000*	36.96 ± 29.33	4.71 ± 11.76	000*	35.72 ± 9.41	2.64 ± 9.14	000*
Mood	42.86 ± 19.07	77.68 ± 20.79	000*	33.93 ± 16.96	83.04 ± 22.62	000*	33.33 ± 19.04	67.71 ± 18.77	000*
Anxiety	35.54 ± 18.12	72.68 ± 25.83	000*	31.82 ± 6.24	71.68 ± 14.89	000*	37.25 ± 11.49	71.13 ± 11.15	000*
Physical domain score	42.66 ± 20.34	85.56 ± 19.43	000*	43.24 ± 19.64	88.66 ± 17.46	000*	42.44 ± 17.88	84.22 ± 19.86	000*
Social domain score	33.46 ± 18.42	78.56 ± 16.54	000*	34.68 ± 17.48	80.26 ± 14.92	000*	32.86 ± 18.24	77.56 ± 16.74	000*
HRQOL 7 days	24.51 ± 14.68	71.42 ± 15.61	000*	22.69 ± 15.66	74.48 ± 15.24	000*	22.46 ± 15.42	70.89 ± 14.98	000*
Overall QOL	22.54 ± 11.64	68.78 ± 19.86	000*	21.89 ± 12.45	71.48 ± 19.88	000*	22.65 ± 12.98	68.64 ± 18.64	000*

**Table 5.** Median overall survival estimate evaluation.

Median overall survival			
Estimate	Std. error	95% confidence interval	
		Lower bound	Upper bound
Arm A 4.0	0.63	2.70	5.30
Arm B 6.0	0.73	4.48	7.52
Arm C 3.5	0.80	1.35	4.65
